# Cooperation between primary cilia signaling and integrin receptor extracellular matrix engagement regulates progenitor proliferation and neuronal differentiation in the developing cerebellum

**DOI:** 10.3389/fcell.2023.1127638

**Published:** 2023-02-21

**Authors:** Anna E. Pittman, David J. Solecki

**Affiliations:** Department of Developmental Neurobiology, St. Jude Children’s Research Hospital, Memphis, TN, United States

**Keywords:** germinal zone, niche, morphogen, integrin, primary cilia, cell polarity, pard complex

## Abstract

Neural progenitors and their neuronal progeny are bathed in extrinsic signals that impact critical decisions like the mode of cell division, how long they should reside in specific neuronal laminae, when to differentiate, and the timing of migratory decisions. Chief among these signals are secreted morphogens and extracellular matrix (ECM) molecules. Among the many cellular organelles and cell surface receptors that sense morphogen and ECM signals, the primary cilia and integrin receptors are some of the most important mediators of extracellular signals. Despite years of dissecting the function of cell-extrinsic sensory pathways in isolation, recent research has begun to show that key pathways work together to help neurons and progenitors interpret diverse inputs in their germinal niches. This mini-review utilizes the developing cerebellar granule neuron lineage as a model that highlights evolving concepts on the crosstalk between primary cilia and integrins in the development of the most abundant neuronal type in the brains of mammals.

## Cerebellar granule neuron introduction

**FIGURE 1:**
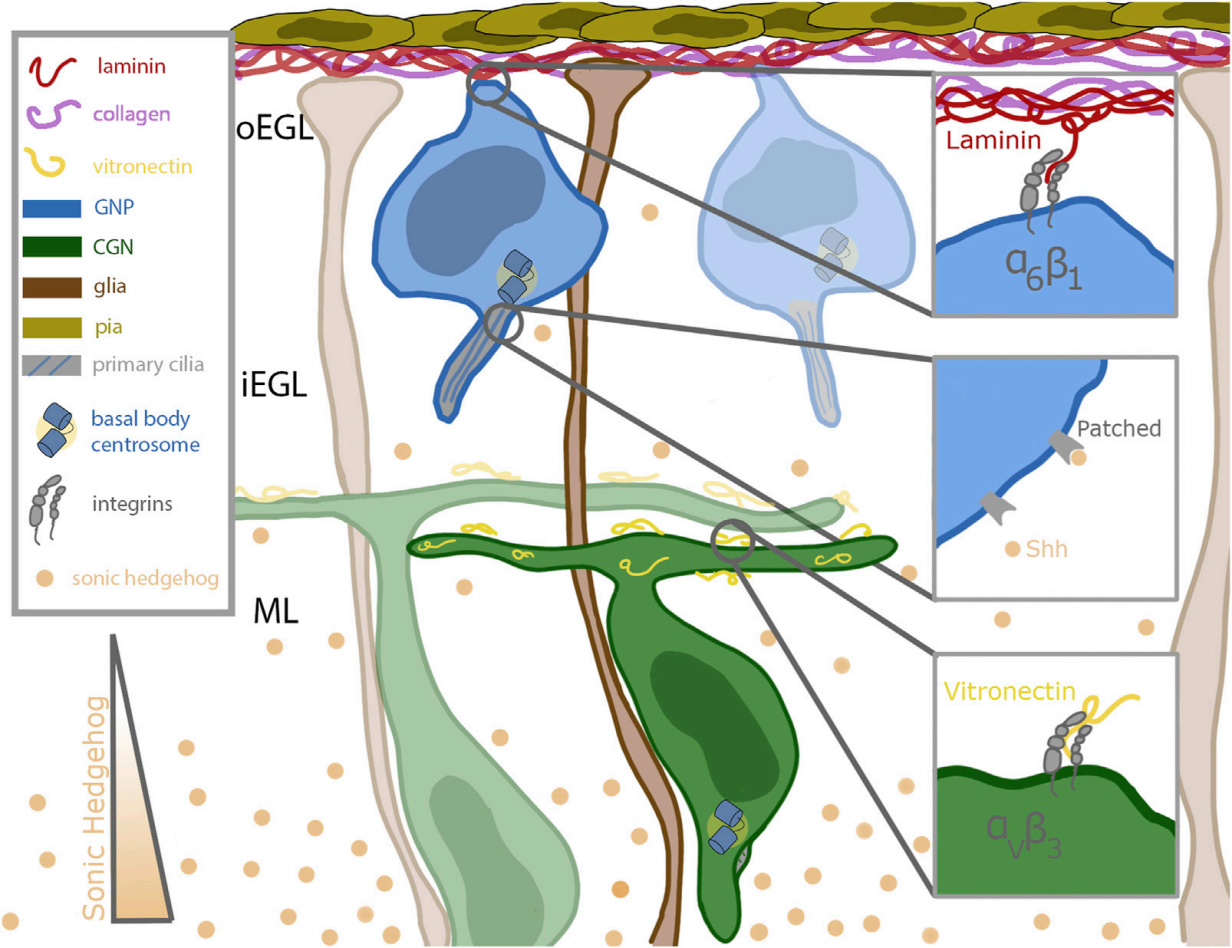
Schematic of cerebellar germinal zone niche. Progenitor cells (shown in blue) are situated near the top of the germinal zone, which is enriched in pia secreted laminin (red) and collagen (purple). The progenitors remain anchored to the laminin through their focal adhesions enriched with alpha 6 beta 1 integrins. Sonic hedgehog (Shh, in tan) binds to the patched receptor which is localized to the primary cilia of the progenitor cell. Shh is secreted by mature differentiated granule neurons and diffuses past the differentiated cells towards the progenitor cells. After differentiation, the granule neurons extend parallel fibers and migrate inward along the bergmann glial fibers (shown in brown) which extend all the way through the germinal zone to the laminin coated basement membrane. The parallel fibers of differentiated neurons are coated with vitronectin (in yellow), a potent signaling molecule which promotes differentiation and blocks ciliogenesis. Vitronectin is secreted by both the Purkinje cells and fully differentiated granule neurons.

The life of a cerebellar granule neuron (CGN) is tightly orchestrated, with temporal and spatial cues that must be carefully followed to ensure the proper acquisition of cell fate and, ultimately, the formation of the cerebellum. CGNs originate in the rhombic lip, and by embryonic day 13.5 (E13.5) they migrate tangentially across the anlage of the developing cerebellum (Alder et al., 1999). As they spread out, they form a second, transitory proliferation germinal zone (GZ) called the external germinal zone (EGL). This germinal zone is subdivided into the outer external granule layer (oEGL) containing granule neuron progenitors (GNPs), which sits adjacent to the basal lamina, and the inner external granule layer (iEGL) containing newly postmitotic CGNs. The GZ and its surroundings contains many extrinsic signals that impinge on GNPs as they undergo a period of clonal expansion to greatly increase their numbers and subsequent differentiation. During their proliferation, GNPs have extensions anchored in the basal lamina (Hausmann and Sievers, 1985) enriched for extracellular matrix components like laminin and collagen (Figure 1). Proliferating CGNs express laminin-specific integrins that help anchor them in the oEGL near the basal lamina-producing pial cells (Blaess et al., 2004). Differentiated CGNs in the iEGL and molecular layer produce an ECM component that decorates the parallel fiber axons called vitronectin, which binds a distinct class of integrin receptors than GNPs in the oEGL (Figure 1). The GZ and areas deeper in the cerebellum, including the molecular layer, also contain high concentrations of the morphogen Sonic Hedgehog (Shh), a mitogen secreted by the Purkinje cells (Wallace, 1999). Shh is a proliferative cue for the GNPs; it binds to the Patched (Ptch) receptor expressed on the primary cilia of the GNPs (Rohatgi et al., 2007) (Figure 1). Deregulation of the proliferation pathways, e.g., through aberrant Shh signaling, disrupts proper GZ exit and has been implicated in GNP transformation in the Shh subgroup of medulloblastoma (Yang et al., 2008). After a proliferative phase that peaks at approximately post-natal day 7 (P7), the progenitors terminally differentiate. During this differentiation, they lose their connection to the basal lamina (Hausmann and Sievers, 1985), become insensitive to Shh, and begin to migrate inwards towards increasing amounts of vitronectin, guided by the Bergmann glial fibers that extend past the Purkinje cell layer (Hatten, 1999) (Figure 1).

A major challenge in developmental neurobiology is to dissect the molecular and cellular mechanisms that developing neurons use to interpret extrinsic signals that modify their differentiation status during the development of neuronal circuity. The CGN is a well-understood model deploying various tools to integrate external cues, two classes of which will be detailed here. An important mitogen sensing tool of the GNP is its primary cilium, which plays a role in the reception of morphogens such as Shh. In contrast, ECM components are sensed by main force-mediating proteins, integrins, critical for proper anchoring in the germinal zone. CGNs express the laminin-specific integrin alpha-6 in the oEGL and lose that expression as they migrate into the iEGL (Pons et al., 2001). As they begin to leave the germinal zone, they express the vitronectin-specific integrin alpha-V (Pons et al., 2001) (Figure 1). Vitronectin, secreted by fully differentiated CGNs, is enriched in the direction of migration (Pons et al., 2001). This minireview will briefly overview the roles of primary cilia and integrins in generalized cell systems and then focus on the developing CGN in detail.

## Introduction to primary cilia and integrins

Primary cilia are non-motile cilia with various functions that are found on almost every eukaryotic cell. Unlike motile cilia, which often coat the cell, primary cilia are generally solitary organelles (Mahjoub, 2013). Initially thought to be a pathologic response to an insult or a non-functional evolutionary holdover (Venkatesh, 2017), their importance was recognized when their absence was correlated with human diseases such as cystic kidney disease (Sharma et al., 2008). Primary cilia serve as chemosensors for mitogens such as those of the Hedgehog (Rohatgi et al., 2007) family and also as mechanosensors by modulating the intracellular calcium concentration in response to fluid shear flow (Praetorius and Spring 2001). Primary cilia consist of a core of nine microtubules in what is referred to as a 9 + 0 arrangement to highlight the lack of the inner two microtubules that only motile cilia possess (in a 9 + 2 arrangement). The microtubule core is sheathed in the cilium membrane, which is continuous with the cell membrane and contains various cilium-specific receptors, such as Patched and Smoothened (Smo) (Corbit et al., 2005; Rohatgi et al., 2007). The primary cilium is anchored to a basal body at its base. The assembly and further elongation of the primary cilium is managed by the intraflagellar transport (IFT) rafts, which are trafficked along the microtubules by kinesin (in anterograde motion along the B-tubule) or dynein (in retrograde motion along the A-tubule) (Reynolds et al., 2018). Ciliogenesis is intrinsically tied to the cell cycle for most cells. SMB55 cells, used to mimic medulloblastoma because of their lack of expression of the Patched receptor, leading to overactivation of the Shh pathway, lose their cilia by the G2 phase of the cell cycle (Ho et al., 2020). Conversely, the cells of some rapidly dividing tumors possess cilia even while in the actively cycling state (Han et al., 2009). The CGN cilium persists well into the early M phase of the cell cycle before being disassembled during late M phase (Ong et al., 2020).

The cell has several pathways through which the length of the primary cilium can be modulated. The length has been shown to be regulated by the IFT protein IFT88 (Rosenbaum and Witman, 2002). Without Kif3a, a subunit of the kinesin motor, primary ciliogenesis does not occur, resulting in stunted primary cilia (Spassky et al., 2008). Additionally chondrocytes can modulate the length of their cilia in response to mechanical load (McGlashan et al., 2010).

Integrins are heterodimeric transmembrane proteins that connect the extracellular matrix (ECM) to the cytoskeleton of a cell. They comprise 18 different alpha subunits and eight different beta subunits, all of which freely diffuse around the cell membrane in a bent-over “off” state. These subunits non-covalently dimerize, giving rise to 24 known distinct combinations, and create complexes with different ligand affinities (Kadry and Calderwood, 2020). Integrin dimers have distinct, but sometimes overlapping, ligand affinities, enabling the cells to respond to complex and spatially varying combinations of ECM substrates, such as laminin, vitronectin, and fibronectin.

Once dimerized, integrins can be activated into an extended “on” state by outside-in signaling, in which a target ligand binds to the dimer, or by inside-out signaling, caused by the binding of an adapter protein to the cytoplasmic tails. Activated integrins bind to other activated integrins to form nascent adhesions, the precursors to mature focal adhesions. Mature focal adhesions are characterized by their association with cytoplasmic adaptor proteins such as talin and vinculin, which link the adhesion to the cytoskeleton (Kanchanawong and Calderwood, 2022). Early studies showed that focal adhesions can undergo remodeling in response to shear fluid force (Davies et al., 1994).

The force exerted by integrins in adhesions can be measured by molecular tension probes, which contain a small ligand that mimics the native substrate of the integrin. These probes make use of Forster resonance energy transfer (FRET) by separating a spectrally matched fluorophore and quencher, by either a deformable linker (polyethylene glycol [PEG] or a DNA hairpin), or on opposite sides of a strand of DNA. A sufficiently large force will either rupture the DNA (with irreversible probes) or extend the linker (with reversible probes) and thus cause an increase in the signal. Reversible probes have been used to investigate the force exerted by integrins in cell types such as a human epithelial cell line (Stabley et al., 2011), human platelets (Brockman et al., 2018), and mouse embryonic fibroblasts (Tan et al., 2020); however, to date, there have been no such investigations of the focal adhesion strength of a CGN.

## Integrins and ECM in the granule neuron lineage

As the main force-mediating complex, integrins play an important role in the ability of CGNs to stay anchored in the germinal zone and to begin migrating towards the Bergmann glial cells. Early electron microscopy studies revealed that proliferating GNPs possess extensions that reach the top of the oEGL and interface with the basal lamina produced by pial cells. Interestingly, these contacts remain in place until the time that GNPs transition to the iEGL, leading the Sievers laboratory to speculate that the basal lamina provides signals that allow GNPs to remain in the proliferative state (Hausmann and Sievers, 1985). Indeed, chemical ablation of the pial that produces the basal lamina leads to premature differentiation and migration out of the GZ (Sievers et al., 1981). Later studies characterized the environment near the pial cells that form the top border of the germinal zone and revealed it to be enriched in the ECM protein laminin (Pons et al., 2001). Proliferating CGNs express integrin beta-1 (ITGB1), which when dimerized with various alpha subunits has a high affinity for laminin. If ITGB1 is knocked out in the CGNs, they lose the ability to sense Shh and to stay anchored in the germinal zone and experience premature germinal zone exit and maturation (Blaess et al., 2004). Accordingly, if the ITGB1 substrate laminin is removed by conditional deletion of laminin from the pial basement membrane, the ability of CGNs to proliferate is diminished (Ichikawa-Tomikawa et al., 2012). The oEGL may be an area of high mechanical signaling in addition to the mitogenic signaling, as GNPs in this area are replete with phospho-FAK 397 phosphorylation, a marker of integrin-based mechanotransduction in many cellular systems (Kullmann et al., 2020). Interestingly, low oxygen tension in the oEGL niche induces ITGB1 expression through the Zeb1 transcription factor (Kullmann et al., 2020), suggesting a complex balance of niche oxygen concentration and mechanical signaling impacts the output of GNP divisions.

Another important ECM protein in the life of a CGN is vitronectin, which is expressed by fully differentiated CGNs. It is found in the iEGL and the IGL, near the Purkinje neurons, and its expression increases as differentiated CGNs increase (Pons et al., 2001). Early studies showed that vitronectin is not essential for development (Zheng et al., 1995); whereas whole-brain laminin depletion results in complete lethality (Alpy et al., 2005), vitronectin-depleted mice developed and survived as normal. Although the pathway is unknown, vitronectin has been shown to stimulate CREB phosphorylation, which has the effect of inducing CGN differentiation (Pons et al., 2001).

As vitronectin presumably plays an important role in promoting migration, it would be interesting to see the effects of removing vitronectin-specific integrins on migration efficiency and cellular morphology. The loss of vitronectin reduced the ability of GNPs to differentiate fully; more of them remained in an early differentiated state, as defined by the expression of TAG1, an axonal glycoprotein expressed in the EGL before migration initiation (Hashimoto et al., 2016). Additionally, vitronectin regulates axon specification during the initial differentiation of GNPs, and its loss hampered axon formation (Oishi et al., 2021).

## Cilia and ciliogenesis in GNPs

**FIGURE 2:**
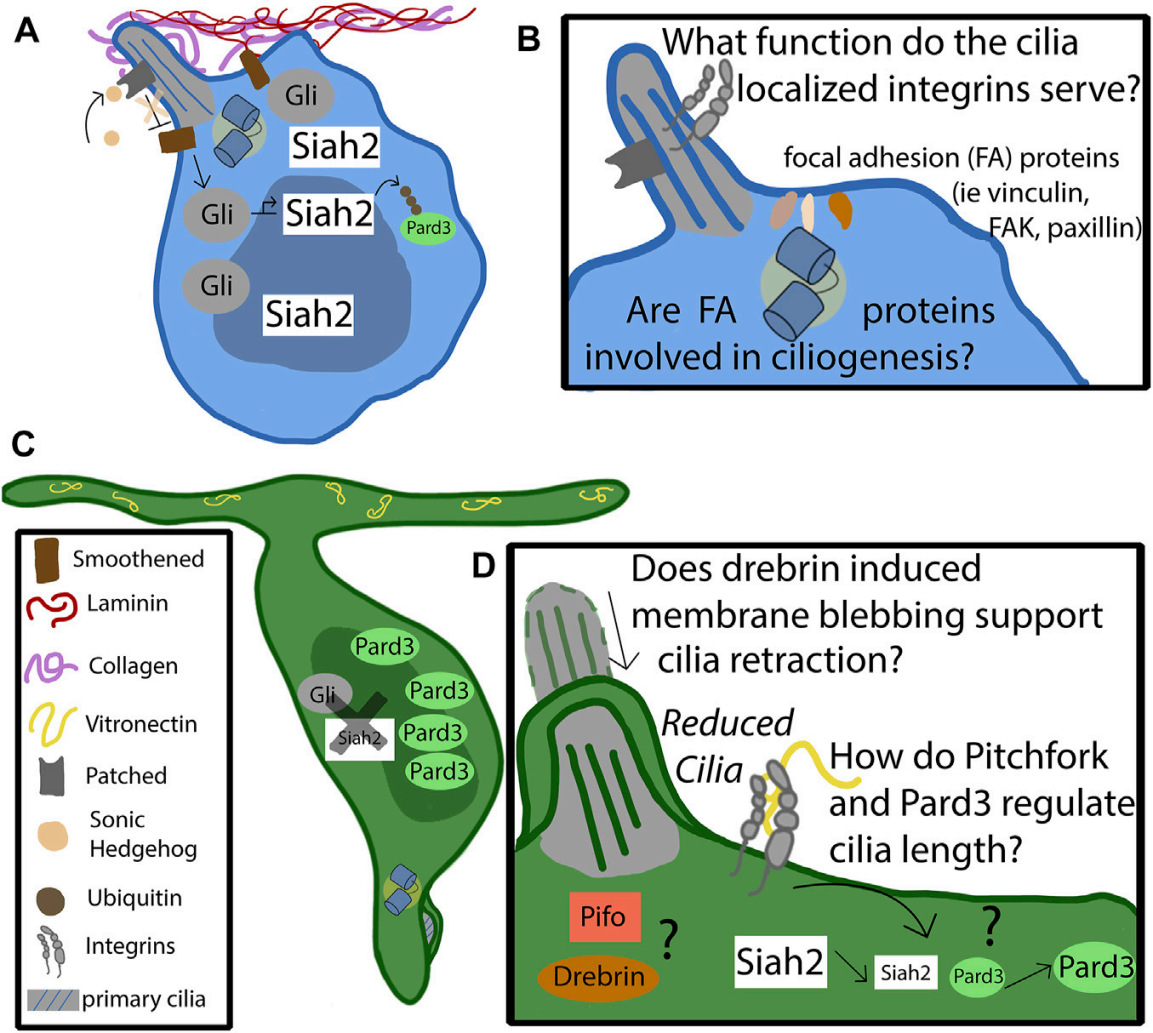
Mechanism of cilia-integrin crosstalk in GNP and CGNs. **(A)** Progenitor cells remain anchored in the germinal zone by adhering to the laminin coated basement membrane through focal adhesions enriched in alpha 6 beta 1. The basement membrane consists of collagen, which surrounds the primary cilia, and laminin, which promotes ciliogenesis. The binding of sonic hedgehog to the patched receptor, localized to the primary cilia, relieves the repression of Smoothened (Smo). Activated Smo serves to activate and stabilize the Gli transcription factors, which upregulate the downstream targets of the hedgehog pathway, such as Siah2. Siah2 tags Pard3 with ubiquitin for degradation. Pard3 is part of the polarity complex and is a driver of differentiation. **(B)** The function of cilia-localized integrins is largely unknown. Do they form canonical focal adhesions, or do they serve as signaling molecules through non-canonical functions? It is known that focal adhesion accessory proteins, such as vinculin, focal adhesion kinase, and paxillin, localize to the basal body at the base of the primary cilia. Are they utilized by the progenitor cell to support ciliogenesis, or to assist in proper positioning of the cilia? **(C)** At some point the feedback loop that promotes the maintenance of the primary cilia destabilizes. The binding of vitronectin to focal adhesions enriched with alpha V beta three causes, through pathways largely unknown, a decrease in Siah2 expression. As Siah2 expression diminishes, Pard3 expression increases and has the effect of repressing the Shh signaling pathway. Primary ciliogenesis is blocked and the cell differentiates and extends parallel fibers. **(D)** The molecular players that cause the cilia to become reduced are largely unknown. Drebrin is known to alter cilia length through membrane blebbing—what role does it play in the granule neuron’s cilia maintenance? Does the cell utilize Pitchfork to regulate the cilia length, as is found in other cell types?

One of the most potent proliferative mitogens for GNPs is the Shh ligand, which is secreted by the Purkinje cells starting at E18.5 (Lewis et al., 2004). Purkinje cells can modulate the release of Shh to increase or maintain the number of proliferating neurons. This helps maintain a favorable ratio of Purkinje cells to CGNs (Willett et al., 2019). Shh binds to the Patched receptor, which is localized to the primary cilia. The binding of Shh to Patched relieves repression of Smoothened, which is a G-protein–coupled receptor that, when activated, can translocate to the cilium (Denef et al., 2000; Varjosalo and Taipale, 2008). Activated Smoothened translates the Shh signal and promotes the translation of full-length and active Gli1–3, three transcription factors that promote the Shh target genes which function to maintain GNPs in both the proliferative and undifferentiated state (Varjosalo and Taipale, 2008) (Figure 2A). Deregulation of the Shh pathway has been implicated in a subgroup of cerebellar medulloblastomas (Yang et al., 2008). What mediates the Shh morphogen response? In studies where IFT88, a protein required for cilium formation, was knocked down, the CGNs struggled to proliferate and experienced premature differentiation (Chizhikov et al., 2007). Thus, primary cilia are a major lynchpin coordinating proliferation and differentiation of GNPs during their massive expansion in the oEGL. At the end of their proliferative cycle, proper GNP germinal zone exit and terminal differentiation of CGNs require GNPs to become insensitive to Shh as differentiating cells are exposed to higher morphogen concentrations as they migrate closer to the source on their journey to the IGL, yet do not muster a proliferative response.

Given the critical role of cilia in controlling GNP proliferation, much effort has been focused on the factors controlling ciliogenesis in this cell type. Recent work shows that the Atoh1 transcription factor essential for GNP specification, CGN lineage survival (Ben-Arie et al., 1997), and medulloblastoma tumorigenesis (Lee et al., 2003), plays a critical role in ciliogenesis (Chang et al., 2019). Atoh1 is expressed in GNPs and protein levels of this transcriptional regulator are normally quickly extinguished as these cells differentiate into CGNs (Akazawa et al., 1995). Atoh1 gain of function (GOF) through *in vivo* misexpression maintains GNPs in the proliferative state and, surprisingly, maintains them in the ciliated state. Indeed, Atoh1 has a causal effect on maintaining cilia as Atoh1 loss of function results in cilia loss and precocious GNP terminal differentiation both *in vitro* and *in vivo*. Epistasis experiments revealed that genetic ablation of cilia *via* Kif3a or Ift88 silencing prevented Atoh1 GOF-induced GNP proliferation, meaning that cilia are necessary for Atoh1 to maintain the GNP state. Indeed, Atoh1 function is required for GNP Shh signaling and Smo translocation to cilia in mouse MB tumor cells (Chang et al., 2019). The deletion of Atoh1 is able to prevent the formation of medulloblastoma, highlighting the important role it plays in regulating cell proliferation (Flora et al., 2009). At a mechanistic level, Atoh1 loss of function leads to improper clustering of centriole satellites, structures associated with cilia essential for ciliogenesis. Combined bioinformatics and expression profiling screening experiments highlighted that centriolar satellite protein Cep131 is not only heavily expressed in GNPs like Atoh1, but its expression is also modulated up and down by Atoh1 GOF and LOF, respectively. Not only is Cep131 a direct transcriptional target of Atoh1 but Cep131 expression rescues ciliogenesis and centriole satellite architecture in Atoh1 deficient GNPs, likely allowing them to respond to Shh morphogen (Chang et al., 2019). Taken together, these results show that GNPs possess a positive cell biological mechanism ensuring ciliogenesis in the oEGL *via* Atoh1 transcriptional activation of its targets.

## Working together: Integrins, ECM control the balance of ciliogenesis and primary cilia loss in CGNs

Despite progress in understanding the pathways that positively regulate ciliogenesis in GNPs, it is still being determined how GNPs lose their ability to respond to Shh as they migrate towards the source of Shh produced in the Purkinje cells. Recent work shows remarkable crosstalk between ECM molecules, integrins, and ciliogenesis in GNPs that weaves GZ intrinsic cues into common pathways controlling GNP differentiation (Ong et al., 2020). The central feature of this crosstalk revolves around the ciliation status of GNPs and CGNs: sophisticated three-dimensional scanning electron microscopy reveals a surprising feature of CGN differentiation. While GNPs are robustly ciliated, as assumed in most somatic cells in mammals, CGNs remarkably have either shortened cilia, no cilia, or cilia located in vesicles where they are not cytoplasmically exposed (Ong et al., 2020) (Figures 2C,D). Using the Fucci cell cycle–specific markers for live cell imaging experiments, GNPs that undergo cell division that produce two daughter cells that remain competent to undergo another round of division (i.e., give rise to two daughter GNPs) maintain their primary cilia until early M phase, disassembling them briefly in late M phase before regaining them in G1. In contrast, the terminally differentiated progeny of GNPs undergoing neurogenic cell divisions do not regrow cilia after M phase, showing CGNs gain the ability to bypass increasing concentrations of Shh by the simple mechanism of diminishing their primary cilia (Ong et al., 2020).

GNP ciliogenesis and lack thereof in CGNs heavily depends on the ECM proteins these cells encounter and, by extension, integrin receptors. Exposing GNPs to a laminin substrate has a synergistic relationship with Shh, in that GNPs are maintained in a proliferative and cilia-bearing state when both signals are present (Blaess et al., 2004). Interestingly when ITGB1, a laminin-specific integrin, is knocked down, GNPs become insensitive to Shh and lose their primary cilia when cultured on laminin. On the other hand, the vitronectin ECM component enriched at the border of the iEGL and distributed throughout the ML has the opposite effect on GNPs: when exposed to Shh and vitronectin, these cells lose their primary cilia and terminally differentiate (Ong et al., 2020). Thus, niche conditions and respective ECM components, like laminin enriched near the basal lamina GNPs maintain contact with or vitronectin located in the iEGL and ML, encode layer-specific spatial signals dictating the competency of GNPs to maintain the primary cilia sensor for the Shh morphogen.

How do laminin and Shh promote the GNP state and ciliogenesis? Integrin-initiated Ras-MAPK pathway activation from laminin substrates and Shh signaling maintain Siah2 E3 ubiquitin ligase expression in GNPs. Siah2 is normally highly expressed exclusively in GNPs and has been previously shown to be necessary and sufficient for GNP GZ occupancy (Famulski et al., 2010). This study reveals a new function of Siah2 as it was also necessary and sufficient for GNP ciliogenesis and Shh signal transduction. Interestingly, Ras-MAPK pathway and Shh signaling maintenance of Siah2 expression represents a unique case of cooperativity between these two previously unconnected signaling cascades. While Shh maintains Siah2 expression in a roughly canonical manner that requires Gli1/2 transcription factors, Siah2 activation also requires both Ras signaling and integrin receptors, showing that Siah2 and its ability to maintain ciliogenesis is a readout of coincidence detection between two key oEGL niche signals. Interestingly, Shh maintenance of Siah2 expression and the feedforward effect on ciliogenesis is reversed when GNPs experience vitronectin-coated surfaces showing how coincidence detection can be subverted simply by altering ECM niche components (Ong et al., 2020) (Figure 2C). Thus, vitronectin is the first known extrinsic signal to have an anti-ciliogenic effect on GNPs and can provide deep insights into how differentiating GNPs ignore such a potent mitogen as Shh.

While Siah2 maintenance of ciliogenesis at first glance bears similarities to Atoh1’s action on cilia, the mechanism is quite different. Siah2 controls GZ occupancy by targeting key proteins, like the Pard3 polarity protein (Famulski et al., 2010) and drebrin microtubule-crosslinker (Trivedi et al., 2017), for ubiquitin-proteasome degradation (Figure 2A). Pard3 is part of the partitioning defective complex, being required for neuronal polarity and promoting germinal zone exit (Solecki, 2022). Thus, Siah2 promotes aspects of the unpolarized GNP state but inhibits the activity of pro-differentiative factors instead of activating the expression of pro-GNP pathways, like Atoh1. Which Siah2 targets contribute towards ciliogenesis? A functional screen showed that Pard3, drebrin, and Pifo could all diminish cilia in the context of Siah2 GOF or elevated Shh signaling, suggesting a model that during GNP differentiation, the amount of Siah2 decreases, leading to an increase in proteins such as Pard3, drebrin, and Pifo and, eventually, to primary cilium disassembly (Figure 2D). Given that vitronectin exposure diminishes Siah2 expression, it plays a critical role in countering the Shh-laminin-Siah2 expression feedback loop to promote GNP differentiation by relieving Siah2 repression of Pard3, drebrin, and Pifo to ultimately block ciliogenesis (Ong et al., 2020).

## Summary and unanswered questions regarding cilia-integrin cooperation

From its birth in the germinal zone near the laminin-producing pial cells, to its decision to migrate towards the vitronectin-secreting Purkinje cells, the neuron is subjected to a cacophony of external and internal cues to guide its journey. CGNs use ITGB1 to stay attached in the germinal zone, and integrins also works through Ras-MAPKs pathway to help support the stability of the primary cilia. The primary cilia enable the neurons to respond to the secreted mitogen Shh and to remain in the proliferative state by binding to the Patched receptor, relieving the repression of Smoothened, and activating the Gli transcription factors. This also corresponds to an increase in Siah2, stabilizing the primary cilia by suppressing Siah2 targets. Vitronectin decreases the expression of Siah2, thus destabilizing the primary cilia and Shh-responsiveness. In the next paragraphs, we discuss the open questions of this new model.

Vitronectin, which is found in increasing concentrations towards the lower border of the germinal zone, counteracts Shh signaling; however, the molecular mechanisms downstream of vitronectin that elicit this effect are still ill-characterized. Shh can bind directly to vitronectin (Pons and Marti, 2000) and may play a role in the exposure of Shh morphogen to the primary cilia. Indeed, vitronectin silencing has been shown to lengthen primary cilia in fibroblast cell lines (Failler et al., 2021). One possible pathway by which vitronectin affects differentiation is through the phosphorylation of PI3k, Akt, and GSK3B. Decreasing vitronectin in dissociated cell cultures led to a decrease in the phosphorylation of these factors and negatively affected the ability of neurons to regulate axon specification, an early stage of differentiation (Oishi et al., 2021). Integrin subunits alpha-V, beta-1, and beta-3 have been implicated in vitronectin binding; knockdown of those subunits decreased the number of CGNs fully differentiating in dissociated cultures (Hashimoto et al., 2016). It would be interesting to investigate how silencing vitronectin-specific receptors effects migration to see if they are necessary and sufficient for diminished Siah2 expression, stabilization of Pard3, and lack of ciliogenesis in CGNs. Recent work in chondrocytes showing that the inhibition of alpha-V, beta-3 integrin dimers lengthens the primary cilia is consistent with the cilia destabilization model. Epistasis analysis with polarity proteins and vitronectin binding integrins will be required to establish a molecular connection further.

As currently conceptualized, the ITGB1 Ras-MAPK signaling model that supports Shh-stimulated Siah2 expressions is agnostic to subcellular localization: It is currently unknown when and where it occurs in the GNPs to support ciliogenesis. Interestingly, there is accumulating evidence that integrin receptors and their downstream interaction partners function near the primary cilia. ITGB1 receptors are known to accumulate in the chondrocyte primary cilia (McGlashan et al., 2006), implying the potential for integrin signaling directly in this organelle. Intriguingly, focal adhesion proteins, that normally intimately associate with integrin-based adhesions, accumulate at the basal body and may anchor cilia to appropriate locations in the cell membrane (Figure 2B). Finally, not only does collagen possess similar activity to laminin to stabilize cilia, but electron microscopy tomography of chondrocyte primary cilia also shows direct association of collagen fibers to primary cilia (Jensen et al., 2004). This accumulating evidence suggests that ciliary integrin signaling is possible but would have to be experimentally addressed with focal inactivation of ciliary integrin receptors. Moreover, the fact that both integrins and cilia are force sensors raises the exciting possibility that ciliary mechanotransduction in cooperation with integrins could regulate Siah2-regulated circuits controlling ciliogenesis. The role of primary cilia as a mechanosensory organelle in the context of CGNs has yet to be explored. Canonically, cilia serve to measure fluid shear stress in various cell types. Does the CGN cilium function in a similar way? Are there measurable differences in intracellular pressure and force between the regions of the developing cerebellum that serve as a guidance cue?
